# Cerebrospinal fluid markers in Creutzfeldt-Jakob disease

**DOI:** 10.1186/1743-8454-5-14

**Published:** 2008-08-27

**Authors:** Anders Skinningsrud, Vidar Stenset, Astrid S Gundersen, Tormod Fladby

**Affiliations:** 1 Multidisciplinary Medical Laboratory, Akershus University Hospital, 1478, Lørenskog, Norway; 2 Department of Neurology, Akershus University Hospital, 1487, Lørenskog, Norway; 3 University of Oslo, Oslo, Norway

## Abstract

**Background:**

The objective was to assess the utility of total tau protein (tTau), the ratio of (tTau)/181 phosphorylated tau protein (P-Tau) and 14-3-3 protein, as diagnostic markers in cerebrospinal fluid (CSF) for Creutzfeldt-Jakob disease (CJD).

**Methods:**

CSF samples received from Norwegian hospitals between August 2005 and August 2007 were retrospectively selected from consecutive patients with tTau values > 1200 ng/L (n = 38). The samples from patients clinically diagnosed with CJD (n = 12) were compared to those from patients with other degenerative neurological diseases: Alzheimer's/vascular dementia (AD/VaD, n = 21), other neurological diseases (OND, n = 5). Total Tau, P-Tau, and β-Amyloid (Aβ_42_) were measured with commercial kits. Additionally, 14-3-3 protein was measured semi-quantitatively by immunoblot.

**Results:**

The minimum cut-off limits for diagnosis of CJD were chosen from the test results. For tTau the lower limit was fixed at 3000 ng/L, for the tTau/P-Tau ratio it was 60, and for 14-3-3 protein it was 0.75 arbitrary units. For tTau and tTau/P-Tau ratio, all but three CJD patients had levels above the minimum, whereas almost all of the other patients were below. For the 14-3-3 protein, two CJD patients were below the minimum and five were above. Only one of the other patients was higher than the limit. The sensitivities, specificities and diagnostic efficiencies were: tTau 75%, 92%, and 87%; tTau/P-Tau 75%, 96%, and 89%; and 14-3-3 protein 80%, 96%, and 91%.

**Conclusion:**

The results suggest that 14-3-3 protein may be the better marker for CJD, tTau/P-Tau ratio and tTau are also efficient markers, but showed slightly inferior diagnostic properties in this study, with tTau/P-Tau marginally better than tTau.

## Background

The prion protein disorder Creutzfeldt-Jakob disease (CJD) is a human transmissible spongiform encephalopathy (TSE) that leads to rapid decay of brain tissue. Human prion diseases are either idiopathic such as sporadic or spontaneous CJD (sCJD), genetic or familial (fCJD), acquired such as iatrogenic CJD (iCJD) or variant CJD (vCJD). Variant CJD is related to bovine spongiform encephalopathy (BSE), has clinical and pathological characteristics different from sCJD and has not been described in Norway. The most common form is sCJD, which accounts for about 85% of all known cases. Spontaneous CJD is a rare fatal disorder with rapid progression, an incidence of approximately 1/million/year [1] and a mortality of more than 90% within one year. The etiology of sCJD is not known, but the pathogenesis is related to conversion of the normal membrane prion protein PrP^c ^to the pathological form PrP^Sc ^[2]. Immunohistochemical demonstration of PrP^Sc ^provides a definite diagnosis of CJD at autopsy or by brain biopsy.

In the Norwegian population of about 4.5 million, one would expect approximately 5 cases of sCJD per year. Due to the few specific pre-mortal diagnostic signs, it is difficult to separate sCJD clinically from rapidly progressing Alzheimer's disease (AD) and other rapidly progressing neurological diseases. As TSEs can cross species barriers, there is a public health concern about the ability of TSE to spread from other species to humans. Classical scrapie in sheep is endemic in Norway, and an atypical variant, Nor 98, has appeared [3], but there is no evidence that scrapie has spread from animals to humans.

The total concentration of tau protein (tTau) in CSF has been found to separate patients with CJD from those with AD [4]. As an increased concentration of tTau is regarded to be a marker for degradation of neurons, the success of tTau as a marker for CJD depends on whether other neurological diseases have the same high amount and rate of neuronal degradation as CJD. A low concentration of the amyloid precursor protein (APP)-derived 42 amino acid peptide in CSF (Aβ_42_), has been correlated with brain amyloid deposition in AD [5]. Elevation of 181 phosphorylated tau protein (P-Tau) is also related to brain pathophysiology in AD, possibly as a result of interaction between amyloid and tau metabolism [6]. As low CSF-Aβ_42 _and elevated P-tau are more specific for AD, one would expect the diagnostic specificity for CJD to rise when the markers are combined. In line with this, the ratio of tTau/P-tau has been described to separate CJD from other neurodegenerative diseases without overlap [7,8]. A separation of sCJD in two clinically different groups according to P-Tau level and a negative prognostic value of elevated P-Tau have been described for CJD [9]. Spontaneous CJD patients with high P-Tau had a shorter disease duration (i.e. they died earlier), had earlier onset of akinetic mutism and a higher incidence of typical EEGs. The occurrence of elevated P-Tau values in CJD will decrease the value of combining tTau and P-Tau in the diagnosis of sCJD because the difference in tTau/P-Tau ratio between the groups would be reduced. The 14-3-3 proteins are evolutionarily conserved proteins present in the cytoplasm of brain neurons at a concentration of about 1% of the total protein content. It has been suggested that 14-3-3 protein as a *pre-mortem *immunoassay marker, may obviate the need for a brain biopsy in the diagnosis of CJD. Although 14-3-3 protein is known to be non-specific, it is included in the WHO criteria for CJD [10].

We wished to investigate the utility of tTau, tTau/P-Tau ratio and 14-3-3 protein measurements in CSF as markers for sCJD. Other diagnostic characteristics for CJD, such as hyper intense magnetic resonance (MR) signals from the basal ganglia, sharp wave complexes in EEG and the examination of the CSF proteins, neuron specific enolase (NSE) and S100 have been evaluated elsewhere [11]. Routine laboratory analyses of the three biological variables, tTau, P-Tau and Aβ_42_, have been offered at the Department of Clinical Chemistry, Akershus University Hospital, since 2003. Routine analysis for 14-3-3 protein has been performed since November 2007. Neurological, psychiatric and outpatient departments in Norway have also had the opportunity to send CSF-samples for analysis.

## Methods

### Patient selection and CSF analysis

CSF samples (n = 691) were received by the Department of Clinical Chemistry, Akershus University Hospital between August 2005 and August 2007 from patients demonstrating symptoms of cognitive decline and possible neurodegenerative disease. This work was supported and approved by the Eastern Norway Regional Health Authority, RHA East, and approved by the Regional Committee for Medical Research Ethics, which also approved an exception from collecting informed patient consent.

Total Tau, P-Tau and Aβ_42 _were analysed in CSF-samples with commercially available enzyme linked immunosorbent assay (ELISA) kits from Innogenetics (Gent, Belgium) adapted to a Tecan Robotic Microplate 150 Processor (Tecan AG, Switzerland). The analyses were performed approximately twice a month. The 14-3-3 protein was analysed by immunoblot with equipment from Invitrogen Corporation Ltd. (Paisley, U.K.) using an antibody against the γ-isoform, anti-14-3-3 gamma, clone CG31-2B (mouse monoclonal IgG_1_, Upstate biotechnology, Lake Placid, NY, USA). The 14-3-3 protein results were assessed semi-quantitatively using arbitrary units. As standard we used dilutions of a homogenate from normal brain. For semi-quantification we performed image analysis using Fujifilm Multi Gauge software (Fujifilm Corporation, Tokyo, Japan). Thirty-nine patients studied retrospectively had tTau values > 1200 ng/L (the highest standard of the kit). One patient was excluded because no additional sample was available to analyse tTau in dilution. Total Tau, P-Tau and Aβ_42 _were measured at the Akershus University Hospital between August 2005 and October 2007. 14-3-3 protein was analysed in seven of the 12 CJD patients and 25 of the 26 AD/VaD/OND patients in October and November 2007 in Akershus University Hospital. In one AD patient and in five CJD patients there was no sample available for analysis. Three of these CJD-patients had qualitative 14-3-3 protein results from laboratories outside Norway. Two CJD patients had no 14-3-3 protein determination. The maximum value for normal clinical values for tTau was 300 ng/L for patients < 50 years, 450 ng/L for patients 50–70 years and 500 ng/L for patients > 70 years [12]. For Aβ_42_, we have previously used values above 450 ng/L for normal levels [13], but after comparison with the Neurochemistry laboratory at Sahlgrenska University Hospital, Gothenburg, Sweden (unpublished data), this has recently been revised to 550 ng/L. The maximum for normal clinical levels for P-Tau was 80 ng/L from the Neurochemistry laboratory in Gothenburg.

### Sample handling

The samples for tTau, P-Tau and Aβ_42 _were initially frozen at -20°C. In February 2006 all old samples were transferred to -80°C and all new samples were kept at -80°C. The samples for 14-3-3 protein were stored for up to 2 years at -80°C before analysis in batches of six. The ELISA standards were run in duplicate and the samples were run singly. The analytical results were read from the corresponding standard curve for each run. No result exceeded the highest standard for P-Tau and Aβ_42_, but for tTau all the patients in this study exceeded the highest standard point of 1200 ng/L. These samples were diluted and rerun.

### Patient diagnosis

The patients were clinically diagnosed according to ICD-10 (International Classification of Diseases -10). The criteria were clinical suspicion, MR findings highly suggestive of CJD and three-phase sharp wave complexes on EEG also considered to be characteristic of CJD. Twelve patients had definite or probable CJD (Table 1); 21 patients had Alzheimer's disease (AD), vascular dementia (VaD), mixed dementia (AD/VaD), frontotemporal dementia (FTD) or unspecified dementia and five patients had other neurological diagnoses (OND, Table 2). Seven of the 12 CJD patients had short disease duration, mean 3.7 months (n = 7, SD = 1.5) and a further two patients had unspecified very rapid progression. Three patients had CJD with slower progression, disease duration 15, 22 and >23 months.

**Table 1:** Characteristics and results for patients with Creutzfeldt-Jakob disease

Sex/age (years)	Disease duration^1^ (months)	EEG	MR	Autopsy	tTau (ng/L)	P-Tau^4^ (ng/L)	tTau/P- Tau (ng/L)	Aβ_42_ ^5 ^(ng/L)	14-3-3 protein
Female/84	2	TSWC^2^	NC^8^	Denied	7,090	49	173	664	Not done
Male/75	6	TSWC	NC	Denied	13,830	211*	65	385*	Positive^7^
Male/62	4	GD^6^	NC	Not done	12,910	149*	83	372*	Not done
Female/58	22	GD	NC	Denied	1,880	92*	26	623	Positive^7^
Female/77	3	TSWC	HSC^9^	Positive^3^	3120	45	69	704	Positive^7^
Female/67	4	TSWC	HSC	No result	8532	66	129	526*	Positive^7 ^0.96
Female/64	Rapid	TSWC	HSC	Positive biopsy ^3^	14,060	84*	167	562	Positive^7 ^3.68
Male/74	2	GD	HSC	Not done	22,460	82*	274	946	2.42
Female/65	>23	TSWC	HSC	Not done	1,343	72	193	M	0.30
Female/70	15	TSWC	HSC	Not done	2,348	93*	25	1051	0.35
Female/61	Rapid	TSWC	HSC	No result	11,038	48	230	755	1.75
Male/54	5	TSWC	HSC	No result	71,900	172*	97	1042	Positive^7 ^9.70

^1^The time from disease recognition to death, ^2^Three-phase sharp wave complexes, ^3^Positive for immunochemical examination of pathological prion protein, PrP^Sc^, ^4^Values above the normal limit used in clinical practice marked with *, ^5^Values below the normal limit used in clinical practice marked with*, ^6^Generalized dysrythmia, ^7^14-3-3 protein found positive in different laboratories outside Norway because some hospitals sent CSF samples for 14-3-3 protein analysis to laboratories outside Norway in parallel to sending samples to us. (Three patients both have semi quantitative results from us and a qualitative, positive result, from a foreign laboratory). ^8^NC, non-specific changes. ^9^HSC, highly suggestive changes of CJD, M = missing value.

**Table 2:** Characteristics, results and diagnosis for patients with Alzheimer's disease, vascular dementia, mixed dementia and other neurological diseases

Sex/age (years)	tTau^1 ^ (ng/L)	P-tau^2 ^ (ng/L)	tTau/ P-Tau	Aβ_42_^3^ (ng/L)	14-3-3 protein^1^ (arbitrary units)	Diagnosis
Female/59	2120	244*	8	401*	M	AD
Female/64	2335	240*	10	347*	0.00	AD
Female/76	1400	220*	6	505*	0.00	AD
Female/79	2443	287*	9	517*	0.00	AD
Female/85	1350	49	27	746	0.59	MCI
Female/73	1380	217*	6	743	0.15	AD
Female/67	1410	199*	7	413*	0.00	AD
Female/62	1670	66	25	348*	0.41	AD/Wernicke
Female/51	1410	27	52	697	0.00	Possible FTD
Female/66	1410	238*	6	622	0.00	MCI/Possible AD
Female/64	1750	49	36	926	0.55	Possible VaD
Male/65	1910	79	24	1297	0.34	Unspecified dementia
Male/65	1280	57	22	1244	0.53	Possible AD/VaD
Female/62	3443	242*	14	312*	0.52	FTD/Possibly AD
Female/67	1540	238*	6	369*	0.00	Probably AD
Female/88	2720	90*	30	464*	0.00	AD/VaD
Male49	1350	81*	17	973	0.00	VaD
Female/72	1780	240*	7	533*	0.00	AD
Female/80	1280	239*	5	699	0.00	Probably AD
Female/76	1480	227*	7	516*	0.00	AD/VaD
Female/76	1360	207*	7	573	0.00	AD
Male/62	8,530*	75	114	1043	0.00	Cerebral lymphoma
Male/71	1,202	59	20	487*	0.00	Cerebral infarction
Female/69	1,992	83*	24	674	0.29	Cerebral infarction
Female/49	1,267	27	47	807	0.95*	Cerebral lymphoma
Female/32	1,433	49	29	960	0.00	Cerebellitis

^1^Values above lower limit for CJD marked with*, ^2^Values above maximum for normal in clinical practice marked with*, ^3^Values below minimum for normal used in clinical practice marked with*, M = missing value.

### Data analysis

Receiver operating characteristic (ROC) curve analysis was performed for tTau and tTau/PTau ratio with the statistical package Analyse-it (Analyse-it Software, Ltd. Leeds, U.K.). ROC-curve analysis was not performed for 14-3-3 protein because some of the results from other laboratories were qualitative only. The AD, VaD, AD/VaD and other dementia patients were treated as one group, AD/VaD. The lower limit chosen for the diagnosis of CJD was 3000 ng/L for tTau, for the ratio of tTau to P-Tau it was 60 and for 14-3-3 protein it was 0.75 arbitrary units. The limits, based on our test results, were set to obtain the best diagnostic performance for each marker. It could be argued that the cut-off for tTau and 14-3-3 protein could have been set slightly higher. In that case for tTau the sensitivity would decrease and the specificity would increase, and for 14-3-3 protein the sensitivity would also decrease and the specificity would increase to 100%. The sensitivity, specificity, positive and negative predictive values, and diagnostic efficiency for the three markers were calculated manually.

## Results

Table 1 presents the characteristics for 12 patients who were diagnosed with CJD, two definite CJD and 10 probable CJD. Eight had MR findings suggestive of CJD. Generalized dysrhythmia and sharp wave complexes on EEG were found in nine patients and generalized dysrhythmia indicating diffuse cerebral pathology in the remaining three. Table 2 presents the characteristics for patients with diagnoses other than CJD. Two patients had VaD or possible VaD, 11 had AD or possible AD, three had AD/VaD or possible AD/VaD, one had AD and Wernicke's encephalopathy, two had possible FTD and two had unspecified dementia. Five patients had OND.

### Total Tau

**Figure 1:**
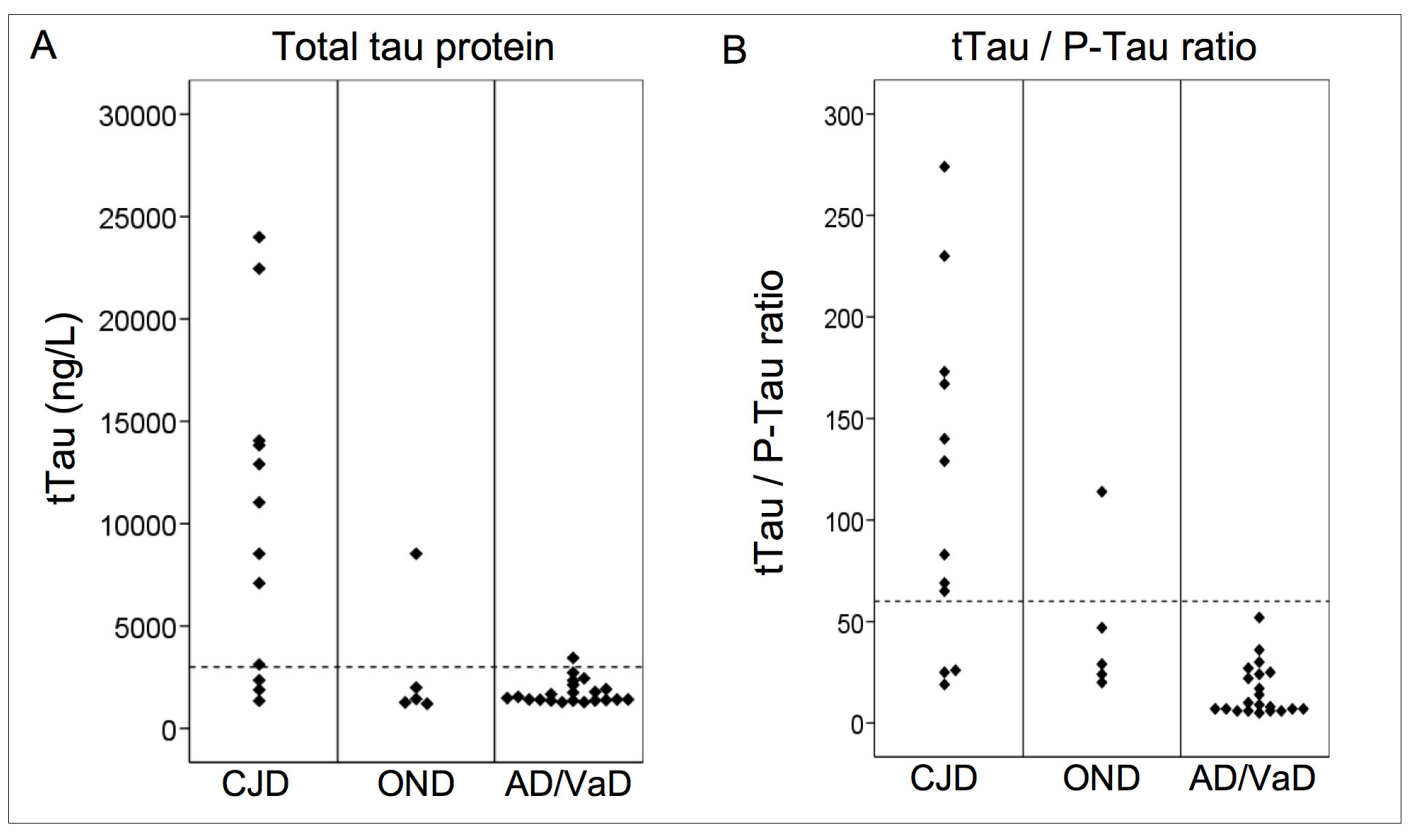
**Data plots of Tau protein (tTau) (A) and tTau/P-Tau ratio (B) by diagnostic group Creutzfeldt-Jakob disease (CJD), other neurological diseases (OND) and Alzheimer's disease or vascular dementia (AD/VaD).** Horizontal dashed lines show chosen lower limit for CJD: tTau: 3000 ng/L, and tTau/P-Tau ratio: 60.

The results for tTau by diagnostic group and lower limits for CJD are presented in Figure 1A. Eleven patients had tTau values above cut-off (3000 ng/L) and 27 below. Nine of the 12 patients with tTau-values above cut-off had CJD, one had OND (cerebral B-cell lymphoma) and one had possible FTD. Three patients with CJD of long duration had values (1343, 1880 and 2350 ng/L) which are below the chosen lower limit, 3000 ng/L. All but one of the OND patients and all but one of the AD/VaD patients had values below 3000 ng/L.

### Ratio of tTau to P-Tau

The results for the ratio tTau/P-Tau by diagnostic group and chosen lower limit for CJD (60) are presented in Figure 1B. Ten patients had tTau/P-Tau ratios above the limit. Nine of these had CJD and one had cerebral B-cell lymphoma. The three patients with CJD of long duration, had values below cut-off (19, 25 and 26). All but one of the OND patients and all AD/VaD patients had values below the limit for CJD.

### P-Tau

**Figure 2:**
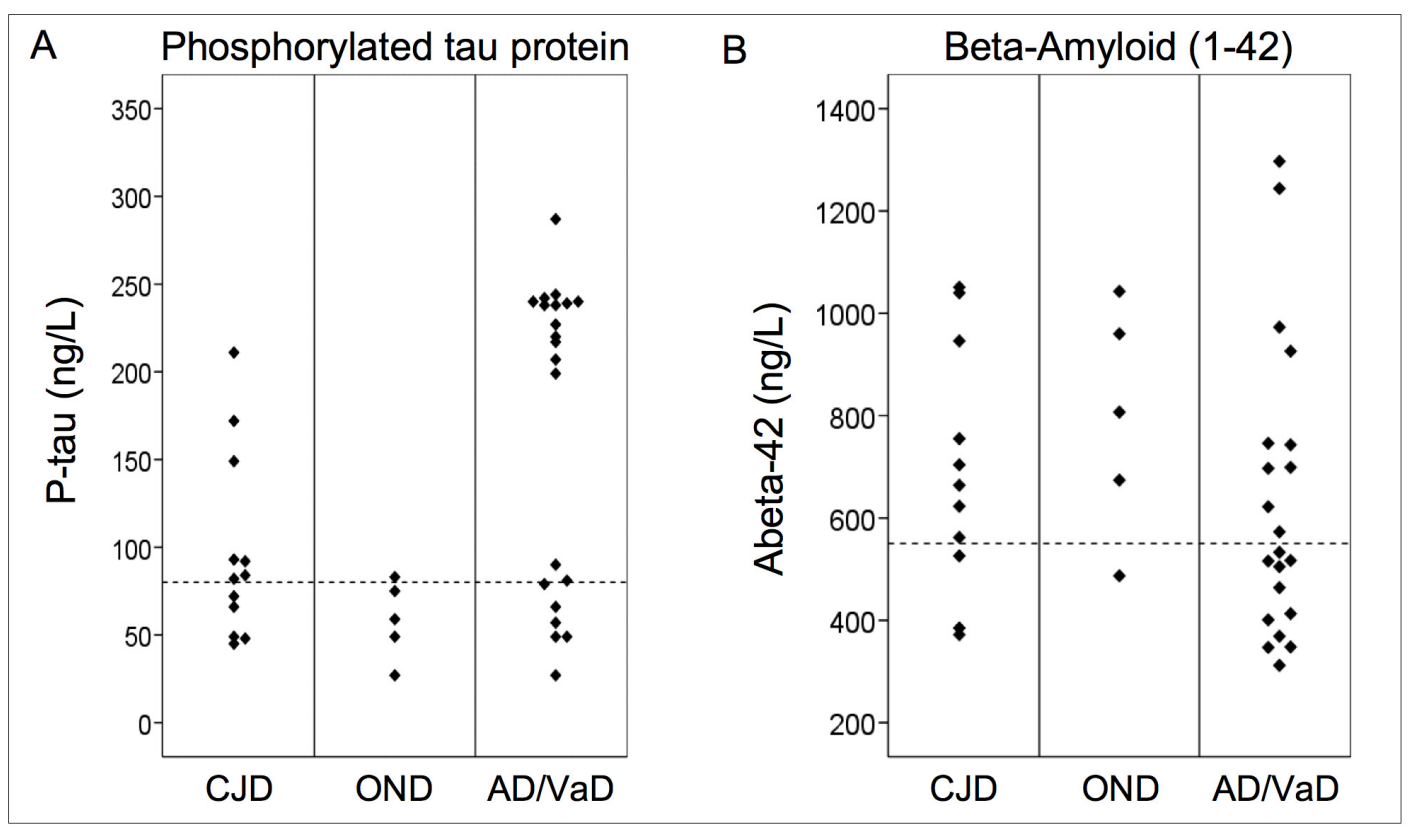
Data plots of 181-phosphorylated tau protein (P-Tau) (A) and beta-amyloid (Aβ_42_) (B) by diagnostic group CJD, OND and AD/VaD with upper (P-Tau) and lower (Aβ_42_) normal limits used in clinical practice (dashed lines): P-Tau: 80 ng/L, Aβ_42_: 550 ng/L.

P-Tau results by diagnostic group and maximum for normal (80 ng/L) are presented in Figure 2A. Six of the 12 CJD patients had P-Tau above normal. In the AD/VaD-group (n = 21) 15 patients (71%) had P-Tau above and six below normal. The P-Tau results in the AD/VaD patients were distributed in two groups, range 27–92 and 200–287 ng/L. One of the OND patients (cerebral infarction) had P-Tau slightly above normal, 81 ng/L.

### Beta amyloid (1–42)

The results for Aβ_42 _by diagnostic group and lower normal limit are presented in Figure 2B. Three of 11 patients (38%) in the CJD group (one missing value) and 11 of the 21 patients (52%) in the AD/VaD group had Aβ_42 _values below normal. The two CDJ patients with the lowest Aβ_42_-values, 372 and 385, also had elevated P-Tau results, 149 and 211 ng/L.

### 14-3-3 protein

**Figure 3:**
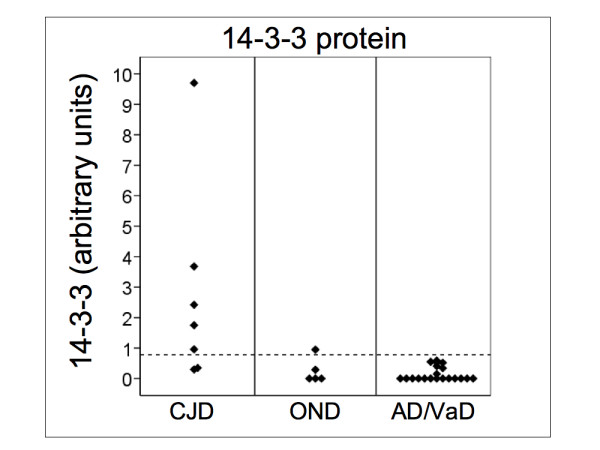
Data plots of 14-3-3 protein by diagnostic group CJD, OND and AD/VaD with lower limit for CJD (dashed line): 0.75 arbitrary units (data from Akershus University Hospital only).

The lower limit for CJD diagnosis was set at 0.75 arbitrary units. Figure 3 shows results from the home laboratory. Samples from 32 patients, seven from the CJD group, all five OND patients and 20 from the AD/VaD group, were available for analysis. Five of the seven CJD patients tested positive. Three of the 14-3-3 positive patients had been tested before and had positive results from laboratories outside Norway. Three of the five CJD patients not tested by us, had been tested before and had positive results. These were included in the estimations of diagnostic parameters (Table 3). Ten of the 12 patients with CJD had been examined for 14.3.3-protein by us, other laboratories or both. Eight of these were positive. One of the five patients with OND (cerebral B-cell lymphoma) tested weakly positive, and all the tested patients in the AD/VaD group were negative. Of the three CJD patients with long disease duration, two were negative.

**Table 3:** A comparison between markers of the diagnostic performance for the diagnosis of CJD.

	**tTau**	**tTau/P-Tau**	**14-3-3 protein**
Sensitivity	75	75	80
Specificity	92	96	96
Positive predictive value	82	90	89
Negative predictive value	89	89	92
Diagnostic efficiency	87	89	91

### Diagnostic performance

Calculations of sensitivity, specificity, positive predictive value, negative predictive value and diagnostic efficiency are presented in Table 3. The ROC curve analysis for the diagnosis of CJD showed that tTau was not significantly different from tTau/P-Tau for the diagnosis of CJD (area under curve 0.897 and 0.918, n.s.).

## Discussion

Although the ROC-curve analysis showed no significant difference between tTau and tTau/P-Tau, the tTau/P-Tau ratio did separate results around the cut-off value more clearly than t-Tau and the specificity and predictive values of a positive test for the CJD diagnosis were slightly better. Thus, our findings seem to agree with those of Riemenschneider *et al *[7], who found that tTau/P-Tau ratio was a better marker for CJD than tTau. Buerger *et al *[14] came to the opposite conclusion, but they measured P-Tau phosphorylated in the 232- position and not in the 181-position as measured here and by Riemenschneider *et al*. Our results suggest that 14-3-3 protein may be a slightly better marker than tTau and tTau/P-Tau.

In contrast to P-Tau, Aβ_42 _did not contribute to providing a more definite diagnosis of CJD. Increased P-Tau was a more specific measure of AD/VaD than low Aβ_42_, and contributed slightly to the diagnostic separation of CJD from the AD/VaD group. Similar values for Aβ_42 _in the CJD and AD/VaD groups suggest that comparable amyloid pathology was present in both groups. This indicates that some CJD patients may have acquired CJD in addition to an increased and possibly AD-related, amyloid pathology. The fact that the two CJD patients with the lowest Aβ_42 _values, also had elevated P-Tau is consistent with an interaction between prion protein and the AD pathological processes [15]. The P-Tau values separated into two groups for AD/VaD patients (Figure 2a). The group with results above 200 ng/L was homogenous clinically because 11 of the 13 patients had the clinical diagnosis of AD, one had FTD/or possibly AD and one had AD/VaD. The group with results below 100 ng/L (n = 8) was clinically more heterogeneous (Table 2). Our results suggest that tTau, tTau/P-Tau and possibly 14-3-3 protein may only be good markers for sCJD of short duration and may not separate the CJD cases with longer duration from the other dementias and OND. This fact may be of considerable importance for the diagnosis of CJD and suggests that brain biopsy should be used more often in dementia cases. In addition, the use of autopsy should be encouraged.

None of the patients in this study were tested for hereditary forms of CJD or AD, and the clinical information did not raise any suspicion about hereditary conditions. Seven patients in the AD/VaD group were <65 years. None of them had a family history of dementia. Our results show that in addition to AD and VaD, other rapidly progressing neurological diseases may have the same tTau and P-Tau biomarker pattern as CJD. One patient with cerebral lymphoma had tTau and tTau/P-Tau above the cut-off values for CJD. Another patient with the same diagnosis was slightly positive for 14-3-3 protein. In spite of using both tTau, P-Tau and 14-3-3 protein for the diagnosis of CJD, our data suggest that there will still be a few patients with AD/VaD and OND that cannot be distinguished from CJD using the biomarkers tTau, P-Tau and 14-3-3 protein. Other investigations will usually establish the diagnosis in these cases by imaging or CSF-cytology.

It can be argued that this study should have been performed prospectively, i.e. patients clinically suspected to have CJD should have been enrolled consecutively. This would have required a more active cooperation between the clinical centres. We were not able to undertake this task at that time, but it should ideally be done as a follow-up of the present study. Starting with high tTau patients was, however, a cost-effective way to find cases. The reason for not analysing 14-3-3 protein at the same time as the other markers was that the analysis was not set up by us until October 2007.

Another criticism that could be raised against our study is the low frequency of histological verification. The reason for this is the right to refuse autopsy and the low autopsy frequency in Norway in general. The importance of obtaining histological verification should be stressed in future prospective CJD studies. Considering the low number of histological verifications, it is possible that some of the CJD patients were wrongly classified. We would argue that this is less likely as eight of the 12 patients (two proven histologically) had MR findings suggestive of CJD. Seven of these also had three-phase sharp wave complexes. Of the remaining four patients, all had non-specific MR changes and two had three-phase sharp wave complexes. The two remaining patients both had non-specific changes on EEG and the disease duration in one of them was four months, highly suggestive of CJD. The other patient had disease duration of 22 months and although 14-3-3 protein was positive, another type of degenerative brain disease cannot be totally excluded.

This study also shows a higher prevalence of CJD in Norway than might be expected. We identified 12 cases during a period of two years. With a prevalence of one case per million inhabitants, approximately 10 cases would be expected, which is quite close to the observed number of 12. However, we do not expect that all the Norwegian cases in this period were known to us. The prevalence of CJD may therefore be higher than anticipated. This indicates a need for closer surveillance of human prion diseases in Norway.

## Conclusion

Total Tau, tTau/P-Tau ratio and 14-3-3 protein are useful, but not entirely sensitive and specific markers for the diagnosis of CJD in CSF. The results indicate that the diagnostic performance of 14-3-3 protein may be slightly better than tTau/P-Tau, which may be slightly better than tTau alone. There is clearly a need for a specific test for CJD, preferably for the misfolded prion protein (PrP^Sc^) itself in readily obtainable biological samples such as blood and CSF. It remains to be seen whether a specific test for PrP^Sc ^would be as sensitive and specific as the markers used in this study.

## Competing interests

The authors declare that they have no competing interests.

## Authors' contributions

AS designed the study, drafted the manuscript, and performed the statistical analysis. VS and TF participated in its design and coordination and helped to draft the manuscript. ASG performed ELISA tests, set up the immunoblot for 14-3-3 protein and contributed to the manuscript. All authors read and approved the final manuscript.
